# Erythematous pink papules following a cat bite

**DOI:** 10.1016/j.jdcr.2024.05.022

**Published:** 2024-05-27

**Authors:** Yousef Salem, Julie Hancock, Alison Messer, Sandra Osswald

**Affiliations:** a Long School of Medicine, San Antonio, Texas; b Department of Dermatology, University of Texas Health Science Center at San Antonio, San Antonio, Texas

**Keywords:** animal bite, atypical mycobacteria, cutaneous infection, immunosuppression, *Mycobacterium chelonae*, *Mycobacterium* infection, nodular lymphangitis, sporotrichosis

## Case

**Fig 1:**
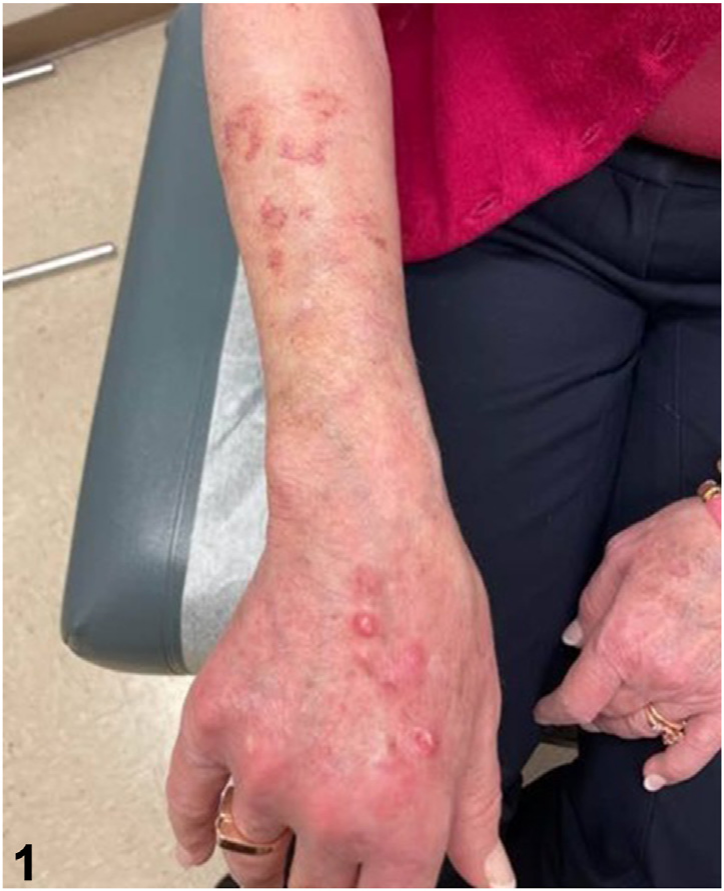

**Fig 2:**
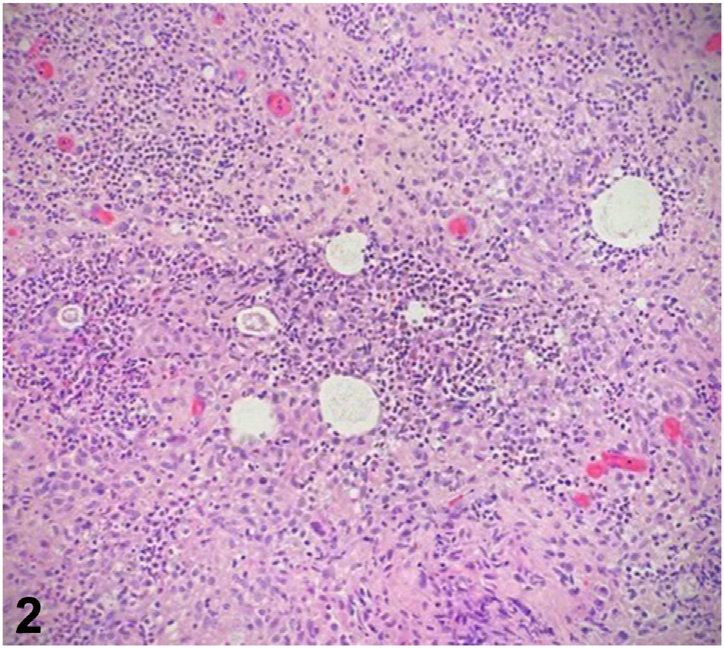

**Fig 3:**
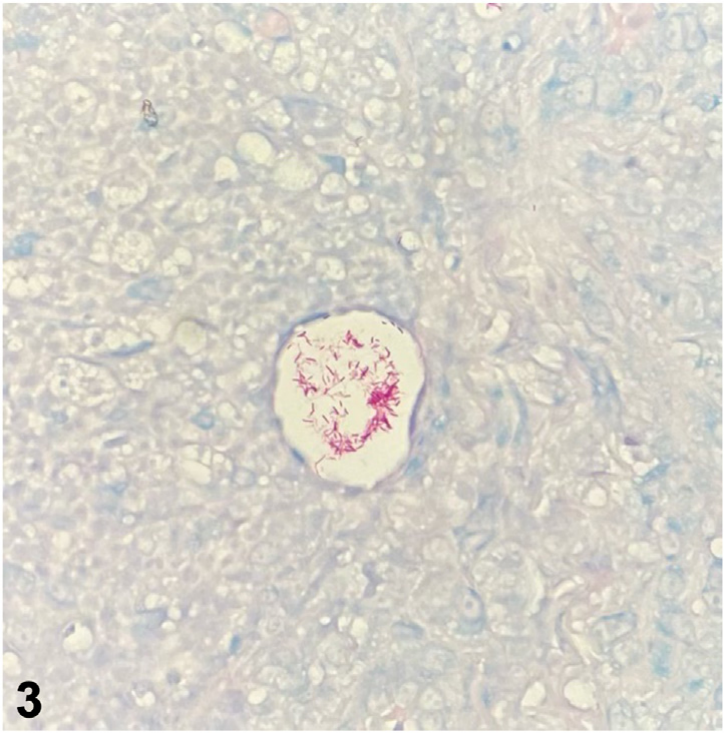

A 71-year-old woman with a history of lupus erythematosus and Sjogren syndrome on hydroxychloroquine, prednisone, and methotrexate presented with tender pink papules and small nodules on her right dorsal hand and forearm (Fig 1) without axillary lymphadenopathy. One month prior, she sustained a cat bite on her right dorsal hand and was treated with a 10-day course of cefpodoxime. The lesions persisted and progressed proximally. She denied recent travel. She received roses from her husband weekly and reported frequent hot tub use. Hematoxylin and eosin stain from a punch biopsy at 20x magnification (Fig 2) and Acid-Fast Bacillus (AFB) special stain are shown (Fig 3).

**Question 1: What is the most likely diagnosis?**

A. Cutaneous lupus
B. Nodular lymphangitis
C. Cat scratch disease
D. Rose Handler’s disease
E. Hot tub folliculitis

**Answers:**

A. Cutaneous lupus – Incorrect. Although this patient has lupus and the distribution is on a photo-distributed location, histology shows a suppurative and granulomatous inflammation. Cutaneous lupus typically shows a vacuolar interface dermatitis with variable lymphocytic inflammation and possible dermal mucin on histology.
B. Nodular lymphangitis – Correct. Nodular lymphangitis is usually associated with a preceding penetrating injury and presents with nodules and papules in the distribution of lymphatic vessels.^1^ The etiology is infectious and potential agents include *Sporothrix schenckii*, atypical mycobacteria, nocardia, and leishmaniasis.
C. Cat scratch disease – Incorrect. Cat scratch disease is typically caused by *Bartonella* species and can present 1 to 3 weeks after inoculation. Tender regional lymphadenopathy is commonly seen in this condition.^2^ Although pathology can be suppurative and granulomatous as seen in this case, *Bartonella henselae* is a gram-negative bacillus that may stain with Warthin-Starry, not an AFB special stain.
D. Rose Handler’s disease – Incorrect. While the patient has frequent exposure to roses and the clinical pattern is of a nodular lymphangitis, the organism in Rose Handler’s disease is *S schenckii*, which are small, elongated yeast that stain with Grocott Methenamine silver or periodic acid-Schiff, not seen on this patient’s histology.
E. Hot tub folliculitis – Incorrect. Although this patient endorsed frequent hot tub use, the typical presentation is of folliculitis in a bathing suit distribution caused by *Pseudomonas* sp. *Pseudomonas* sp. are gram negative rods and may be seen by gram stain but not typically on AFB stain.

**Question 2: Which of these organisms is the most likely cause of this presentation?**

A. *Staphylococcus aureus*
B. *Mycobacterium chelonae*
C. *Pasteurella multocida*
D. *Pseudomonas aeruginosa*
E. *S* *porothrix* *schenckii*

**Answers:**

A. *Staphylococcus aureus* – Incorrect. *S aureus* colonizes the skin and is a common cause of skin infection. However, it is typically seen as gram positive cocci on histology.
B. *Mycobacterium chelonae* – Correct. *M chelonae* and other atypical mycobacteria can cause nodular lymphangitis and a Fite stain would demonstrate numerous acid-fast bacilli as was seen in this case.^1^ Tissue culture was further required to specifically identify *M chelonae*, which may be part of felines’ oral flora depending on their diet. This patient’s chronic immunosuppression likely made her more susceptible to infection; however, this presentation is possible even in immunocompetent patients.
C. *Pasteurella multocida* – Incorrect. *P multocida* is the most common bacteria associated with cat bites but does not typically stain with an AFB stain.^3^
D. *Pseudomonas aeruginosa* – Incorrect. *P aeruginosa* cutaneous infections have a variable presentation ranging from green nail syndrome to folliculitis to cutaneous necrotic eschars when systemic. This typically is a gram-negative organism that is negative on AFB staining.
E. *Sporothrixschenckii* – Incorrect. Although this patient has a history of exposure to roses and *Sporothrix* can be a cause of nodular lymphangitis, the organism is typically difficult to find on histology. It appears as a small elongated yeast which may stain with GMS or periodic acid-Schiff but is negative on AFB stain.

**Question 3: What treatment would be most appropriate for this patient?**

A. Rifampin and isoniazid for 6 months
B. Cephalexin for 10 days
C. Fluconazole for 6 weeks
D. Azithromycin and doxycycline for 4 months
E. Ciprofloxacin for 2 weeks

**Answers:**

A. Rifampin and isoniazid for 6 months – Incorrect. Rifampin and isoniazid are more appropriate for the treatment of latent tuberculosis. While rifampin does have activity against some atypical mycobacterium, *M chelonae* is unlikely to respond well to this antibiotic combination.^1^
B. Cephalexin for 10 days – Incorrect. Cephalexin is a first-generation cephalosporin with limited activity against atypical mycobacterium. It is more often used to target staphylococcal or streptococcal infections.^3^
C. Fluconazole for 6 weeks – Incorrect. Fluconazole is an antifungal agent that is not effective against mycobacterium species.
D. Azithromycin and doxycycline for 4 months – Correct. Combination therapy with a macrolide antibiotic and tetracycline for a long-term period (at least 3 months) are appropriate to target *M chelonae.*^1^ Ideally, antibiotic treatment is based on specific susceptibilities if available. The use of doxycycline over other tetracyclines such as minocycline minimizes the risk of severe cutaneous adverse reactions.
E. Ciprofloxacin for 2 weeks – Incorrect. Fluoroquinolones alone are inappropriate for the treatment of this atypical mycobacterium and would need to be combined with a macrolide or tetracycline to account for resistance.^1^

## Conflicts of interest

None disclosed.
